# Persistent C-peptide secretion in Type 1 diabetes and its relationship to the genetic architecture of diabetes

**DOI:** 10.1186/s12916-019-1392-8

**Published:** 2019-08-23

**Authors:** Paul M. McKeigue, Athina Spiliopoulou, Stuart McGurnaghan, Marco Colombo, Luke Blackbourn, Timothy J. McDonald, Suna Onengut-Gomuscu, Stephen S. Rich, Colin N. A. Palmer, John A. McKnight, Mark W. J. Strachan, Alan W. Patrick, John Chalmers, Robert S. Lindsay, John R. Petrie, Sandeep Thekkepat, Andrew Collier, Sandra MacRury, Helen M. Colhoun

**Affiliations:** 1 Usher Institute of Population Health and Informatics, University of Edinburgh, Old Medical School, Teviot Place, Edinburgh EH8 9AG, UK; 2 Institute of Genetics and Molecular Medicine, University of Edinburgh, Western General Hospital Campus, Crewe Road, Edinburgh, UK; 3 Institute of Cardiovascular and Medical Sciences, University of Glasgow, Glasgow, UK; 4 Royal Infirmary of Edinburgh, Edinburgh, UK; 5 Metabolic Unit, Western General Hospital, Edinburgh, UK; 6 Diabetes Centre, Victoria Hospital, Kirkaldy, UK; 7 David Matthews Diabetes Centre, Monklands Hospital, Airdrie, UK; 8 Center for Public Health Genomics, University of Virginia, Charlottesville, USA; 9 Medical School, University of Exeter, Exeter, UK; 10 Medical School, University of Dundee, Dundee, UK; 11 Glasgow Caledonian University, Glasgow, UK; 12 NHS Highland Diabetes Centre, Inverness, UK

**Keywords:** C-Peptide, Diabetes mellitus type 1, Age at diagnosis, Insulin secretion, Genetics, Cross-sectional studies

## Abstract

**Background:**

The objective of this cross-sectional study was to explore the relationship of detectable C-peptide secretion in type 1 diabetes to clinical features and to the genetic architecture of diabetes.

**Methods:**

C-peptide was measured in an untimed serum sample in the SDRNT1BIO cohort of 6076 Scottish people with clinically diagnosed type 1 diabetes or latent autoimmune diabetes of adulthood. Risk scores at loci previously associated with type 1 and type 2 diabetes were calculated from publicly available summary statistics.

**Results:**

Prevalence of detectable C-peptide varied from 19% in those with onset before age 15 and duration greater than 15 years to 92% in those with onset after age 35 and duration less than 5 years. Twenty-nine percent of variance in C-peptide levels was accounted for by associations with male gender, late age at onset and short duration. The SNP heritability of residual C-peptide secretion adjusted for gender, age at onset and duration was estimated as 26%. Genotypic risk score for type 1 diabetes was inversely associated with detectable C-peptide secretion: the most strongly associated loci were the HLA and *INS* gene regions. A risk score for type 1 diabetes based on the HLA DR3 and DQ8-DR4 serotypes was strongly associated with early age at onset and inversely associated with C-peptide persistence. For C-peptide but not age at onset, there were strong associations with risk scores for type 1 and type 2 diabetes that were based on SNPs in the HLA region but not accounted for by HLA serotype.

**Conclusions:**

Persistence of C-peptide secretion varies widely in people clinically diagnosed as type 1 diabetes. C-peptide persistence is influenced by variants in the HLA region that are different from those determining risk of early-onset type 1 diabetes. Known risk loci for diabetes account for only a small proportion of the genetic effects on C-peptide persistence.

**Electronic supplementary material:**

The online version of this article (10.1186/s12916-019-1392-8) contains supplementary material, which is available to authorized users.

## Background

Studies using sensitive assays for C-peptide have shown that some degree of residual insulin secretion commonly persists for more than 5 years after diagnosis of type 1 diabetes [1–4]. However, few studies have examined the frequency with which C-peptide secretion persists after long duration of type 1 diabetes or across a wide range of ages of onset. Using an assay with lower limit of detection 17 pmol/l, the prevalence of detectable C-peptide in non-fasting serum samples was reported to be much lower (6% versus 78%) in those diagnosed in childhood with long (> 40 years) duration than in those diagnosed as adults with short duration (3–5 years) [3]. In another study, fasting C-peptide at diagnosis was found to be lower and to decline more steeply with time in those with younger age at onset [5].

Nonetheless at any age of onset or duration, there is variation in C-peptide persistence, the determinants of which are poorly understood but may be partly genetically determined. One recent genome-wide association study in 3479 people with type 1 diabetes, but mostly diagnosed in childhood, identified a locus on chromosome 1 and multiple variants in the HLA region associated with C-peptide levels adjusted for sex, age at diagnosis and diabetes duration [6].

The objective of this study was to investigate the relationship of detectable C-peptide secretion to clinical features and to the genetic architecture of diabetes in a population-based cohort of people with a clinical diagnosis of type 1 diabetes spanning a wide range of age at diagnosis and duration. A specific objective was to test whether heterogeneity in C-peptide persistence in people clinically diagnosed as type 1 was explained by inclusion of misdiagnosed cases of type 2 diabetes or by genetic heterogeneity in cases of type 2 diabetes.

## Methods

### Study population

The Scottish Diabetes Research Network Type 1 Bioresource (SDRNT1BIO) is a cohort of people clinically diagnosed as type 1 diabetes aged 16 years and older at recruitment. Questionnaire data and samples obtained on the day of recruitment were linked to clinical data from the Scottish Care Information Diabetes Collaboration electronic health record [7]. The cohort comprises one third of the adult population with type 1 diabetes in Scotland and its representativeness has been described in detail [8]. In Scotland, most people diagnosed with diabetes do not have auto-antibodies measured at diagnosis, and the clinical diagnosis of type 1 is based on age at diagnosis, time to insulin, any history of ketoacidosis, and exclusion of monogenic subtypes of diabetes.

Of 6127 people recruited into the study, there were 6076 with a clinical diagnosis of type 1 diabetes or latent autoimmune diabetes of adulthood after excluding those diagnosed with monogenic subtypes of diabetes (intentionally recruited for the cohort) or diabetes from other causes. Median age at onset was 21 (interquartile range 12 to 31) years, and median duration of diabetes at enrolment was 21 (interquartile range 11 to 31) years. In 120 of these individuals, more than 1 year had elapsed from diagnosis to starting insulin, ascertained from prescription records and questionnaire responses.

### Laboratory measurements

Non-fasting serum samples were obtained at clinic visit in 5928 of those clinically diagnosed as type 1. The median time from sampling to freezing at − 80^∘^C was 2 h 15 min (interquartile range 1 h 30 min to 3h 10 min). Plasma glucose measured in these blood samples was greater than 5 mmol/l in 88% of individuals. Non-fasting random C-peptide levels in people with type 1 diabetes are highly correlated with C-peptide levels after a mixed meal [9]. C-peptide measurements on these samples were undertaken at the Exeter Clinical Laboratory using the Roche electrochemiluminescence assay [10], with a lower limit of detection of C-peptide of 3 pmol/l. Autoantibodies to glutamic acid decarboxylase (GAD65), tyrosine phosphorylase-related protein 2 (IA2) and zinc transporter 8 (ZnT8) were measured at the Exeter laboratory, which participates in the Diabetes Antibody Standardisation Programme [11].

Antibody titres exceeding the 97.5th percentile of the reference range were scored as positive. The 97.5th percentiles for GAD and IA2 are 11 and 7.5 World Health Organization (WHO) units/ml respectively. For ZnT8, the 97.5th percentile was 65 WHO units/ml in those aged up to age 30 years and 9.1 in those aged more than 30 years. Those with at least one antibody level above the reference range were classified as autoantibody-positive. These autoantibody measurements were used in combination with C-peptide measurements used to identify possible misdiagnosed cases of type 2 diabetes. The rationale for this was that in those who have residual beta-cell function as indicated by high C-peptide levels, we would expect autoantibodies to be still present if diabetes were caused by autoimmmune beta cell damage. This classification based on C-peptide levels and autoantibody status was validated by examining genotypic scores as described in the “Results” section.

### Genotyping

The cohort was typed with the Illumina Human Core Exome 24 1.0 chip at the Center for Public Health Genomics, University of Virginia. After qualitychecks, genotypes were available on r of those clinically diagnosed as type 1 diabetes. Genotypes were phased and imputed to the UK10K reference panel with the EAGLE algorithm [12], and the imputed genotypes were filtered to exclude SNPs with minor allele frequency less than 0.02 or proportion of information extracted less than 0.7.

### Calculation of genotypic scores from summary statistics

Genotypic risk scores for type 1 diabetes and type 2 diabetes were computed using the GENOSCORES platform described elsewhere [13]. Univariate regression coefficients from publicly available meta-analyses [14, 15] were supplemented with single-SNP scores for additional type 1 diabetes-associated loci reported in a further meta-analysis from which only one SNP per locus was published [16]. Other meta-analyses that did not give the magnitudes and signs of the effects could not be used to calculate scores. Diabetes-associated SNPs were filtered at a threshold *p* value of 10^−5^. Locus-specific scores were generated for regions containing at least one SNP with *p* value less than 10^−6^ and separated from other filtered SNPs by a gap of at least one megabase. All other filtered SNPs were combined into a residual genome-wide score. The threshold *p* value used to designate a genomic region as a diabetes-associated locus limits the number of regions thus designated but does not make any difference to the genome-wide score.

The GENOSCORES platform adjusts the locus-specific scores for linkage disequilibrium between SNP genotypes by premultiplying the vector of univariate SNP coefficients, obtained from summary GWAS results, by the generalized inverse of the correlation matrix between these genotypes. This correlation matrix was estimated from the 1000 Genomes European ancestry reference panel. The relative weights of the SNPs obtained by this procedure approximate the weights that would be obtained by fitting a multivariate regression model to the individual-level data. In principle, this method should capture additive effects across each genomic region, but not interaction effects between alleles at the same locus (dominance) or different loci (epistasis).

After restricting to SNPs that were contained in the type 1 Bioresource genotype dataset, this procedure generated 41 locus-specific scores for type 1 diabetes and 60 locus-specific scores for type 2 diabetes. There were five risk loci that were common to both types of diabetes—*BMP8A*, HLA region, *CENPW*, *ASCC2* and *BCAR1/CTRB1/CTRB2*. Although the HLA region is not generally considered an established risk locus for type 2, it was included as a locus-specific score based on the criterion of at least one SNP with *p* value less than 10^−6^. For each diabetes type, locus-specific scores and the residual genome-wide score were summed over loci to obtain the full genome-wide score.

For type 1 diabetes, separate scores were constructed for HLA serotypes and for other SNPs in the HLA region. HLA serotypes at the DQB1 and DRB1 loci were imputed from the untyped SNPs using the HIBAG program [17] with reference serotypes based on all European ancestry individuals in the 1000 Genomes panel [18]. Alleles at these loci were grouped as follows: 0301 to 0304 at the DRB1 locus as DR3, 0401 to 0413 at the DRB1 locus as DR4 and 0302 to 0305 at the DQB1 locus as DQ8. Serotypes at these two loci were classified into six groups—DR3/DR4-DQ8, DR3/DR3, DR4-DQ8/DR4-DQ8, DR4-DQ8/X, DR3/X and X/X—to which score weights were assigned as published by Oram et al. [19]. The HLA region-specific polygenic score for type 1 diabetes was regressed on this HLA serotype score, and the residuals from this regression were included in the analysis as the “HLA residual” score. The HLA region was excluded from the genome-wide score for type 2, so that the type 2 score could be used to discriminate between liability to type 2 and liability to type 1.

Each locus-specific score was scaled to unit standard deviation so that effect sizes could be compared. The genome-wide genotypic scores were standardized to have zero mean and unit standard deviation in White British participants without diabetes in UK Biobank.

#### Comparison with genotypic scores in UK Biobank participants

To validate the classification of diabetes type in the SDRNT1BIO cohort, we compared the distributions of genotypic scores in these groups with the genotypic scores in UK Biobank participants with and without diabetes whose self-reported ethnicity was White British. Of these participants, 16,427 reported that they had been diagnosed with diabetes (excluding those diagnosed only with gestational diabetes). One thousand four hundred thirteen of these were categorized as type 1 diabetes based on questionnaire report that they had been diagnosed before age 50 years and started insulin within a year of diagnosis, and the remaining 15,014 were categorized as type 2 diabetes.

### Statistical analysis

For modelling associations of clinical covariates, age at onset was transformed by taking the square root, and C-peptide levels were transformed by taking the logarithm to base 10, and setting the log transform of values below detection threshold to zero. As preliminary analysis showed that these associations varied with age at onset, interaction terms with age at onset were included in these models.

To allow for relatedness, the relationship matrix was computed from the unimputed genotypes and the R package *GMMAT* [20] was used to fit linear mixed models for age at onset and for C-peptide. The model for C-peptide was adjusted for sex, age at onset and duration. Fitting this linear mixed model yields an estimate of heritability and allows genome-wide SNP association tests to be computed efficiently from the gradient of the log-likelihood (efficient score) at the null.

## Results

### Relation of C-peptide secretion to age at onset, duration and auto-antibody status

**Fig. 1:**
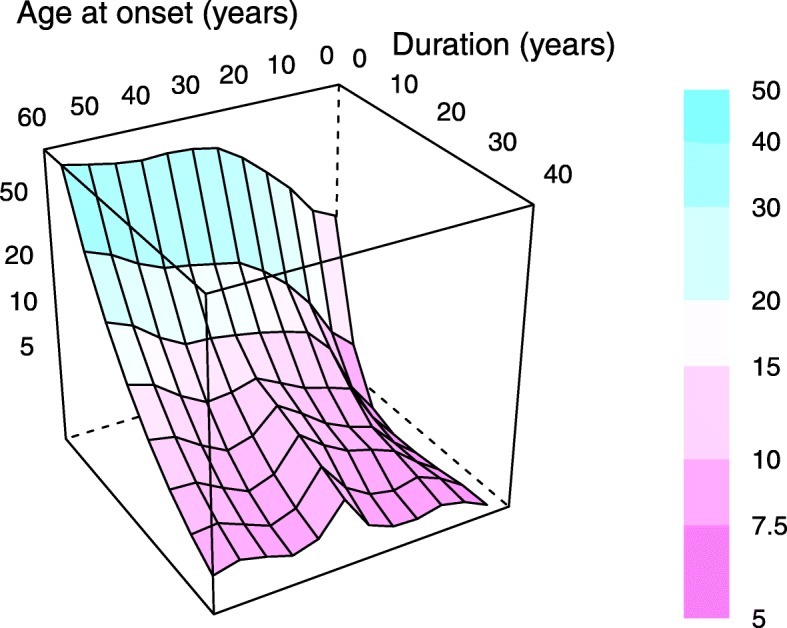
Geometric mean plasma C-peptide (pmol/l) by age at onset and duration. A smoothed fit of log C-peptide to age at onset and duration was computed by LOESS regression of polynomial degree 2 and span 0.25, then evaluated over a grid of values of the predictor variables. The C-peptide level (pmol/l) of each panel is encoded both as its colour and as its coordinate on the vertical axis

Table 1 shows the frequency of detectable C-peptide by age at onset and duration among those classified clinically as type 1 diabetes. Prevalence of detectable C-peptide levels varied from 19% in those with onset before age 15 and duration greater than 35 years to 72% in those with onset after age 35 and duration less than 15 years. For C-peptide levels above 50 pmol/l—a threshold previously associated with better glycaemic control [21]—the prevalence rates in the same groups were respectively 4% and 58%. Geometric mean C-peptide levels by age at onset and duration (smoothed by LOESS regression) are shown in Fig. 1.

**Table 1:** Prevalence of detectable C-peptide by age at onset and duration (years)

Age at onset	0 to 15	15 to 25	25 to 35	35–
Duration				
0 to 5	76% 13/17	96% 178/186	94% 161/172	92% 262/285
5 to 10	62% 79/128	67% 110/165	78% 107/138	74% 188/253
10 to 15	25% 60/237	55% 89/163	52% 103/197	57% 129/225
15 or more	19% 317/1643	26% 259/996	36% 253/705	42% 174/418
All	23% 469/2025	42% 636/1510	51% 624/1212	64% 753/1181

**Table 2:** Proportion negative for autoantibodies by duration (years) and C-peptide (pmol/l)

C-peptide range	[0, 30]	(30, 200]	(200, 600]	(600, 7e+03]
Duration				
0 to 5	5% 5/92	9% 15/168	12% 28/243	35% 54/155
5 to 10	15% 49/326	13% 26/196	28% 24/87	69% 47/68
10 to 15	17% 97/568	21% 26/122	45% 25/56	75% 53/71
15 or more	30% 997/3272	36% 108/300	59% 68/116	80% 49/61
All	27% 1148/4258	22% 175/786	29% 145/502	57% 203/355

Table 2 shows that most people who had C-peptide levels above 600 pmol/l and had been diagnosed at least 5 years earlier were autoantibody-negative. Accordingly, the 203 individuals with C-peptide greater than 600 pmol/l who were negative for all three autoantibodies were classified as “possible type 2”, and all others as “definite type 1”.

**Fig. 2:**
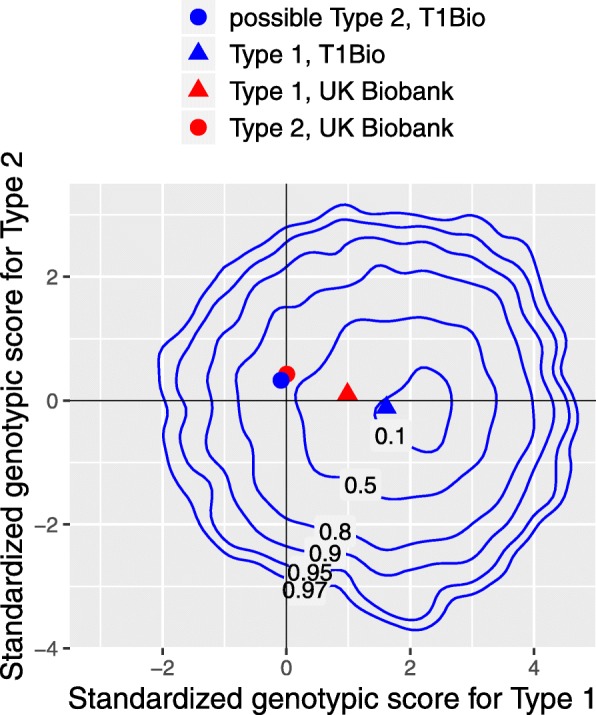
Joint probability contours of genotypic scores for type 1 and type 2 diabetes excluding those classified as possible type 2. Labels show probability enclosed by each contour. Scores standardized to zero mean and unit standard deviation in UK Biobank White British participants without diabetes

To examine whether the genetic risk profile of the possible type 2 individuals was more similar to that of people with type 2 than type 1 diabetes, we plotted the contours of the joint probability distribution of the genome-wide risk scores for type 1 and type 2 diabetes in those classified as definite type 1 diabetes (Fig. 2). The mean of the type 1 genotypic risk score in the cohort is about 1.6 standard deviations above the mean for White British UK Biobank participants without diabetes. The mean genotypic risk scores for type 1 and type 2 in the cohort members classified as possible type 2 were close to the mean for UK Biobank participants classified as type 2. On this basis, those classified as possible type 2 were excluded from the further analyses reported below.

### Heritability and associations with clinical covariates and genome-wide genotypic risk scores

#### Age at onset

The SNP heritability of age at onset (transformed to the square root and adjusted for gender) was estimated as 0.3. Age at onset was associated inversely with genotypic risk score for type 1 diabetes (standardized regression coefficient -0.23 yr^0.5^, *p*= 10^-50^). However, gender and genotypic risk scores accounted for only 4% of the variance of age at onset.

#### C-peptide

The heritability of C-peptide (log-transformed and adjusted for gender and age at onset × duration) was estimated as 0.26. In a linear regression model with log C-peptide as dependent variable, 29% of variance in log C-peptide was explained by age at onset, duration and gender. As early age at onset was associated in this cohort with female sex (coefficient with square root of age as dependent variable − 0.17), some of the association of female sex with lower C-peptide levels was explained by age at onset.

Table 3 summarizes a regression model with C-peptide as dependent variable and plasma glucose (dichotomized at 5 mmol/l), age at onset, gender, duration, genotypic risk scores and body mass index as covariates. Terms for interaction of age at onset with other covariates are included in this model. To make the results easier to interpret, covariates are centred to have zero mean so that each main effect represents the predicted effect of that variable when other covariates are at their mean values. C-peptide levels were not associated with plasma glucose levels. C-peptide levels were associated positively with age at onset and higher genotypic score for type 2 diabetes and inversely with female gender, duration and genotypic score for type 1 diabetes (Table 3). The effects of gender, duration and body mass index were dependent upon age at onset, and the signs of the coefficients (main effect and interaction effect in the same direction) show that these effects were stronger in late-onset than in early-onset cases.

**Table 3:** Regression of log C-peptide on gender, age at onset, duration, glucose, body mass index and genotypic risk scores for diabetes, excluding those with possible type 2 defined by autoantibody status and C-peptide level

	Estimate	Std. error	*p* value
Plasma glucose > 5 mmol/l	0.0066	0.029	0.8
$\sqrt {\text {age onset}}$	0.11	0.0083	2e −40
Female gender	−0.074	0.022	0.001
Duration (years)	−0.03	0.00093	2e −206
Type 1 DM risk score	−0.04	0.0088	5e −06
Type 2 DM risk score	0.024	0.0089	0.007
Body mass index (kg/m^2^)	0.0031	0.0024	0.2
$\sqrt {\text {age onset}} \times $ gender	−0.079	0.015	1e −07
$\sqrt {\text {age onset}} \times $ duration	−0.0089	0.00059	2e −51
$\sqrt {\text {age onset}} \times $ Type 1 score	−0.0087	0.0058	0.1
$\sqrt {\text {age onset}} \times $ Type 2 score	0.0042	0.0059	0.5
$\sqrt {\text {age onset}} \times $ BMI	0.0044	0.0017	0.008

### Associations of age at onset and C-peptide with SNP genotypes

**Fig. 3:**
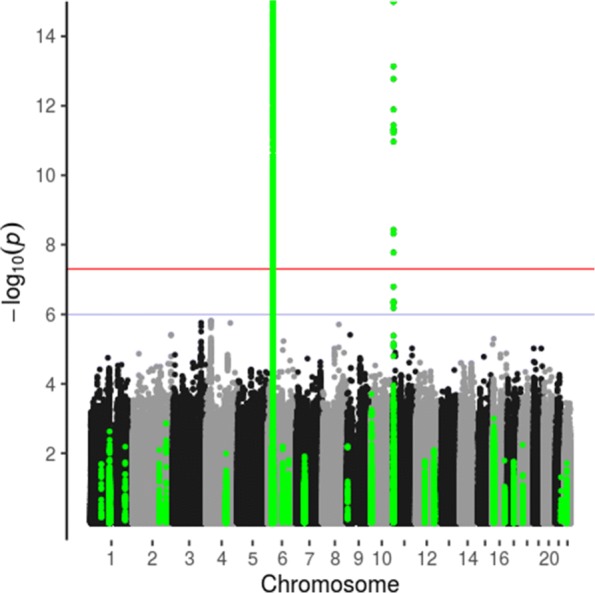
Manhattan plot of genome-wide association study of age at onset (vertical axis truncated at 15). Type 1 diabetes-associated regions in green

**Fig. 4:**
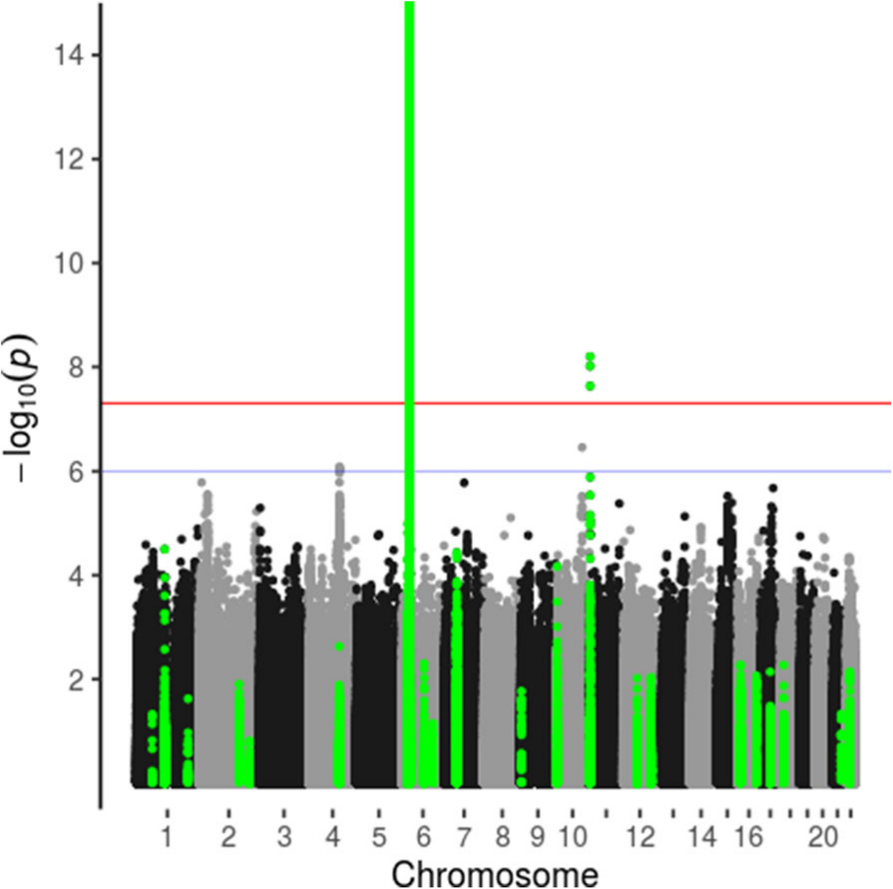
Manhattan plot of genome-wide association study of C-peptide levels, adjusted for age at onset, duration and predicted age at onset given genotype (vertical axis truncated at 15). Type 1 diabetes-associated regions in green

Genome-wide associations of age at onset and C-peptide levels with SNP genotypes are summarized in Manhattan plots in which regions containing diabetes-associated SNPs (based on those included in the genotypic scores) are highlighted (Figs. 3 and 4). The regression models for age at onset included sex as covariate, and regression models for C-peptide levels included sex, age at onset, duration and age at onset × duration. The only SNP associations with age at onset and C-peptide for which the *p* values were less than 10^−7^ were in the HLA and *INS* gene regions. The reported association of the *PTPN22* 185T variant (rs2476601) with C-peptide persistence [22] was confirmed (Table 4). Reported associations of SNPs outside the HLA and *INS* regions with age at onset [23] or associations of SNPs in the HLA region with C-peptide but not age at onset [6] were not confirmed in this study (Table 4). The SNP rs1983890 near the PFKFB3 gene, previously reported to be associated with latent autoimmune diabetes of adulthood [24], was not associated with age at onset or with detectable C-peptide secretion (Table 4).

**Table 4:** GWAS results for age at onset and for C-peptide adjusted for sex, age at onset and duration: associations with SNPs previously reported

Chr	SNP	Position	Alleles	Freq	Score age onset	*p* value age onset	Score C-peptide	*p* value C-peptide
1	rs2476601	114377568	A/G	0.83	9.4	0.7	160.8	3e −04
6	rs72975913	128293932	A/C	0.85	−36.9	0.1	−16.9	0.7
10	rs1983890	6178614	T/C	0.66	−28.9	0.4	−32.4	0.6
6	rs9260151	29911030	T/C	0.91	−39.1	0.04	−100.6	0.003
6	rs1264813	29939900	T/C	0.91	96.6	3e −07	123.3	2e −04
6	rs61211515	30100975	C/CT	0.87	79.2	3e −04	107.3	0.005
6	rs3135002	32668439	A/C	0.96	−124.2	6e −20	−258.3	3e −28
1	rs559047	238753916	A/T	0.77	−16.9	0.6	−37	0.5

Sources for reported association: rs2476601 (C1858T variant in *PTPN22*) [22], rs72975913 [23], rs1983890 [24], all others [6]

### Associations of age at onset and C-peptide with locus-specific genotypic risk scores

**Fig. 5:**
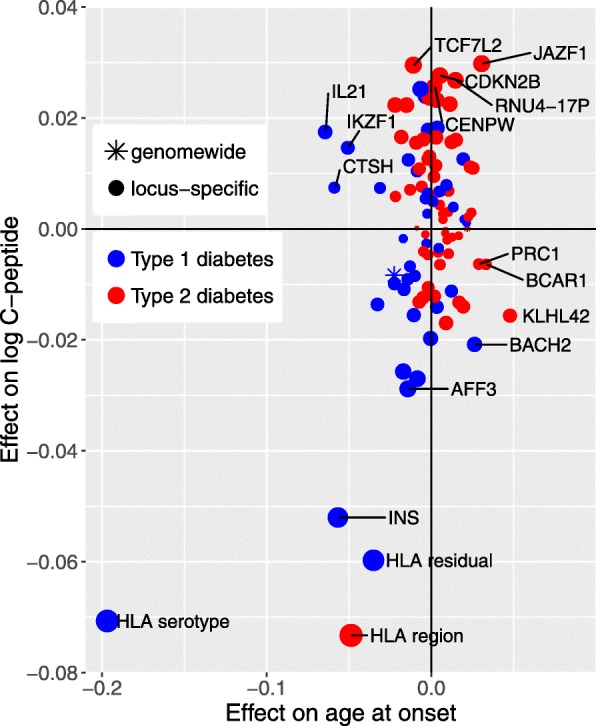
Locus-specific genotypic scores for type 1 or type 2 diabetes: plot of univariate effects on log10 C-peptide (adjusted for age at onset and duration) against univariate effect on square root age at onset. Effect sizes are the effect of a change of one standard deviation in each locus-specific score

In Fig. 5, the effects of locus-specific genotypic scores are displayed by plotting the regression slope for C-peptide (adjusted for gender, age of onset and duration) against the regression slope for age at onset. The regression coefficients and *p* values are given in Additional file 1: Tables 5, 6, 7 and 8. The HLA residual score for type 1 diabetes, calculated by adjusting the HLA region-specific polygenic score for HLA serotype score, represents the residual contribution of the HLA region to risk of type 1 diabetes that is not explained by HLA serotype.

This plot shows that by far the strongest genetic effect on age at onset is that of HLA serotype. Other type 1 diabetes-associated regions that contribute are the *INS*, *IL21* and *CTSH* gene regions. Scores for type 2 diabetes were not in general associated with age at onset.

For C-peptide persistence, the strongest effect was that of the HLA score for type 2 diabetes. Several locus-specific scores for type 2 diabetes, including *JAZF1* and *TCF7L2*, are associated with C-peptide persistence. For a few loci, the effects on age at onset and the effects on C-peptide levels appear to be discrepant in direction or size. Thus, the *IL21* score for type 1 diabetes was strongly associated with early age at onset but not with low C-peptide level. The *KHL42* score for type 2 diabetes was associated with late age at onset but not with high C-peptide levels. The score for the *CTSH* gene region, in which SNP genotypes were previously reported to be associated with clinical remission at 1 year from diagnosis in children with type 1 diabetes [25], was strongly associated with age at onset but not with C-peptide persistence.

Of the five loci that were common to both types of diabetes, there were four—*BMP8A*, HLA region, *CENPW*, *ASCC2*—for which scores for type 1 and type 2 diabetes were positively correlated in the type 1 Bioresource implying that the same alleles or haplotypes are associated with increased risk of both types of diabetes. For the *BCAR1/CTRB1/CTRB2* region, the scores were negatively correlated (*r* = − 0.20), consistent with studies showing that the G allele of the rs720877 SNP in this region is associated with increased risk of type 1 diabetes but with decreased risk of type 2 diabetes [14, 15, 26].

## Discussion

### Frequency of detectable C-peptide secretion

The high prevalence of detectable C-peptide in this cohort is consistent with most other recent studies [3, 4] that have used highly sensitive assays. Of the 585 patients aged less than 15 years at onset and with at least 35 years duration, 85% had no C-peptide detectable with this assay. Although detectable C-peptide secretion is common, for those diagnosed in childhood, however, our results do not support the assertion that “the majority of patients with long-duration Type 1 diabetes are insulin microsecretors” [2]. Although we did not use a mixed meal to stimulate C-peptide secretion, plasma glucose measured on the same samples (dichotomized at 8 mmol/l) was not associated with C-peptide level in a regression model. Comparisons of the prevalence of detectable C-peptide secretion between studies are difficult to interpret because these studies differ in distribution of age at onset, gender, criteria for type 1 diabetes and detection limits of the assays used.

If this cross-sectional population-based sample represents the natural history of C-peptide persistence in type 1 diabetes, we can infer that C-peptide falls rapidly within the first 10 years in early-onset cases, but more slowly in late-onset cases, continuing to decline more than ten years after diagnosis. A similar pattern was reported in a European multicentre study [5]. This is consistent with cross-sectional results reported previously for individuals diagnosed after 11 years of age [27]. An unexpected finding was that in late-onset cases, C-peptide levels appear to fall off more rapidly in women than in men.

The clinical picture labelled as latent autoimmune diabetes in adults [28, 29], in which islet antibodies are present but insulin is not required during the first 6 months after diagnosis [30, 31], may simply be one end of a spectrum from classic juvenile-onset type 1 diabetes to later-onset cases with slowly progressive loss of beta-cell function [32, 33]. The absence of auto-antibodies does not necessarily exclude autoimmune pathogenesis of diabetes even in people with high C-peptide levels [34]. However, we have validated our classification of type 1 and possible type 2 diabetes by comparing the mean genotypic risk scores of these categories in the SDRNT1BIO cohort with the distributions of those who can be reliably classified as type 1 or type 2 in the UK Biobank cohort. Further confirmation that we have managed to exclude misdiagnosed cases of type 2 is that age at onset is not associated with genotypic risk score for type 2 in those we have classified as type 1. A practical implication is that in late-onset cases presenting with a clinical picture that is compatible with type 1 diabetes, autoantibody testing might be useful in excluding cases of type 2 diabetes who will not need long-term insulin therapy. We have noted that summary GWAS results for type 1 and type 2 diabetes show at least four risk loci where the same alleles are associated with increased risk of both types, implying some overlap in aetiologies.

### Genetic associations with age at onset and C-peptide persistence

Other studies of genetic associations with age at onset and C-peptide persistence in type 1 diabetes have tested individual SNPs for association as in a conventional GWAS [6, 22, 23]. We have undertaken a GWAS but have also taken a different approach, using publicly available summary GWAS results to compute genotypic risk scores for each diabetes-associated genomic region. Because the prior hypothesis space is far smaller than it would be in a conventional GWAS, this approach makes it possible to detect effects that would be missed in a conventional GWAS of individual SNPs.

As C-peptide persistence depends on age at onset, a first step in understanding the genetic architecture of C-peptide persistence is to study genetic effects on age at onset. Others have reported [35] that average genetic risk scores for type 1 diabetes are lower in late-onset than in early-onset cases of type 1 diabetes. To estimate the relative mixture proportions of type 1 and type 2 on the assumption that the distribution of genotypic risk scores in type 1 diabetes is independent of age at onset [36] is likely to underestimate the proportion of type 1 cases. In contrast to a recent meta-analysis of 15696 cases typed with the ImmunoChip [23] which found strong evidence for association with age at onset in only two genomic regions—the HLA region and the *PTPRK* / *THEMIS* region on 6q22.33—we find that several other risk loci for type 1 diabetes are also associated with age at onset. The strongest effect on age at onset of any region outside the *HLA* region is the *IL21* gene region, which has a relatively small effect on risk of type 1diabetes itself.

Because C-peptide persistence is related to age at onset, to detect specific genetic effects on C-peptide secretion, it is necessary to adjust for age at onset. Although the SNP heritability of residual C-peptide adjusted for gender, age at onset and duration is 26%, genetic risk scores for diabetes explain only an additional 1% of variance in this adjusted phenotype and most of the remaining genetic and environmental variance remains unexplained. By adjusting the polygenic score for type 1 diabetes in the HLA region for HLA serotype at the *HLA-DRB1* and *HLA-DQB1* loci, we were able to distinguish the effects of serotype from the effects of other genes in the HLA region. The strongest effects on age at onset (as on type 1 diabetes itself) are from HLA serotype, but for C-peptide, the strongest effects are those of variants in the HLA region that are independent of HLA serotype and are associated with increased risk of type 1 and type 2 diabetes. To investigate further the effects of the HLA region on C-peptide persistence that are distinct from the effects of this region on risk of juvenile-onset diabetes, it will be necessary to model the joint effects of HLA genes in case-control comparisons combined with case-only studies of C-peptide persistence. We note also that some risk loci for type 2 diabetes are associated with C-peptide persistence but not with age at onset: this implies that they influence the clinical phenotype of late-onset type 1 diabetes even though they do not influence the risk of developing this condition.

## Conclusions

Persistence of C-peptide secretion varies widely in people clinically diagnosed as type 1 diabetes. Known risk loci for diabetes account for only a small proportion of the genetic effects on C-peptide persistence. C-peptide persistence is influenced by variants in the HLA region that are different from those determining risk of early-onset type 1 diabetes. Further exploration of how genetic effects on C-peptide persistence differ from the established genetic effects on islet-cell autoimmunity may provide insights into pathways that could be targeted to limit or reverse the loss of beta cell function in type 1 diabetes.

## Additional file

Additional file 1 **Table 5.** Regressions of age at onset on regional genotypic scores for type 1 diabetes. **Table 6.** Regressions of age at onset on regional genotypic scores for type 2 diabetes. **Table 7.** Regressions of log C-peptide on regional genotypic scores for type 1 diabetes. **Table 8.** Regressions of log C-peptide on regional genotypic scores for type 2 diabetes. (PDF 34 kb)

## Data Availability

The aggregate-level data objects together with the Rmarkdown script that generated this manuscript are available from the corresponding author upon request. In accordance with governance requirements, a data access committee oversees applications for access to individual-level data as described elsewhere [8].

## Abbreviations

GAD65: Glutamic acid decarboxylase
GWAS: Genome-wide association study
HLA: Human leukocyte antigen
IA2: Tyrosine phosphorylase-related protein 2
SDRNT1BIO or T1Bio: Scottish Diabetes Research Network Type 1 Bioresource
WHO: World Health Organization
ZnT8: Zinc transporter 8
