# Supplementary material for: Persistent C-peptide secretion in Type 1 diabetes and its relationship to the genetic architecture of diabetes
Source: BMC Med. 2019 Aug 23;17:165. doi: 10.1186/s12916-019-1392-8 (PMC6706940; doi:10.1186/s12916-019-1392-8)
Supplement: Supplementary file 1 — Table 5. Regressions of age at onset on regional genotypic scores for type 1 diabetes. Table 6. Regressions of age at onset on regional genotypic scores for type 2 diabetes. Table 7. Regressions of log C-peptide on regional genotypic scores for type 1 diabetes. Table 8. Regressions of log C-peptide on regional genotypic scores for type 2 diabetes. (PDF 34 kb) [file 12916_2019_1392_MOESM1_ESM.pdf]

Table 5 Regressions of age at onset on regional genotypic scores for type 1 diabetes

Chrom	Start (Mb)	End (Mb)	Num SNPs	Nearest gene	Coefficient	*p* value
1	39.526	39.585	2	*BMP8A*	−0.002	0.9
1	63.643	63.66	3	*PGM1*	−0.009	0.5
1	113.286	113.994	28	*PTPN22*	−0.008	0.5
1	172.706	172.71	1	.	−0.003	0.8
1	206.767	206.784	2	*IL10*	−0.003	0.8
2	19.704	19.704	1	.	−0.013	0.3
2	100.13	100.254	10	*AFF3*	−0.014	0.3
2	162.254	162.404	4	*IFIH1*	−0.011	0.4
2	203.829	203.93	13	*CTLA4*	−0.002	0.9
4	*122.078*	*122.64*	*9*	*IL21*	*−0.064*	*1e−06*
4	165.65	165.653	1	.	0.012	0.4
5	168.799	168.808	1	*SLIT3*	0.019	0.1
6	*25.082*	*33.885*	*941*	HLA residual	*−0.035*	*0.008*
6	*25.082*	*33.885*	*941*	HLA serotype	*−0.197*	*1e−50*
6	90.104	90.293	7	*BACH2*	0.026	0.05
6	126.334	126.556	3	*CENPW*	0.004	0.8
7	*50.399*	*50.971*	*6*	*IKZF1*	*−0.051*	*1e−04*
9	4.283	4.296	8	*GLIS3*	−0.033	0.01
10	6.038	6.14	13	*IL2RA*	−0.017	0.2
10	33.13	33.137	1	*NRP1*	0	1
11	*2.08*	*2.291*	*13*	*INS*	*−0.057*	*2e−05*
11	119.966	119.966	1	.	−0.022	0.09
12	55.971	56.125	12	*ERBB3*	−0.017	0.2
12	110.922	112.755	38	*SH2B3*	−0.017	0.2
14	68.797	68.797	1	.	−0.017	0.2
14	98.033	98.033	1	.	0.021	0.1
15	*78.939*	*78.947*	*2*	*CTSH*	*−0.059*	*9e−06*
16	10.946	11.342	36	*CLEC16A*	0.003	0.8
16	28.29	28.62	8	*IL27*	−0.01	0.4
16	75.213	75.262	3	*BCAR1*	−0.031	0.02
17	39.82	40.668	13	*ORMDL3*	0.009	0.5
17	45.997	45.997	1	*MAPT*	−0.004	0.8
18	12.747	12.877	8	*PTPN2*	0.02	0.1
18	69.863	69.872	2	*CD226*	−0.014	0.3
19	46.716	46.716	1	*PRKD2*	−0.014	0.3
19	48.704	48.704	1	*FUT2*	0.004	0.7
20	1.63	1.695	4	*RN7SL561P*	0.001	0.9
20	9.424	9.424	1	*PLCB4*	−0.007	0.6
21	42.416	42.428	5	*UBASH3A*	−0.002	0.9
22	29.715	30.765	17	*ASCC2*	0.005	0.7
22	37.148	37.195	3	*RAC2*	0.013	0.3
.	.	.	29	residual genome-wide	−0.022	0.09

Table 6 Regressions of age at onset on regional genotypic scores for type 2 diabetes

Chrom	Start (Mb)	End (Mb)	Num SNPs	Nearest gene	Coefficient	*p* value
1	39.147	39.57	6	*BMP8A*	− 0.001	0.9
1	213.972	213.99	15	*PROX1*	0.025	0.06
1	219.479	219.615	14	*ZC3H11B*	0.004	0.7
2	43.222	43.623	67	*LINC01126*	− 0.009	0.5
2	60.307	60.372	17	*MIR4432HG*	− 0.013	0.3
2	65.415	65.457	3	*SPRED2*	− 0.002	0.9
2	160.234	160.4	18	*ITGB6*	0.019	0.1
2	226.133	226.317	17	.	0.005	0.7
3	11.986	12.372	14	*ACTG1P12*	− 0.018	0.2
3	22.936	23.441	4	*RPL24P7*	− 0.007	0.6
3	64.714	64.744	7	*ADAMTS9-AS2*	− 0.007	0.6
3	123.217	123.391	7	*ADCY5*	0.007	0.6
3	185.556	185.83	20	*IGF2BP2*	0.003	0.8
4	6.263	6.323	31	*PPP2R2C*	0.012	0.4
4	94.08	94.215	9	*HMGB3P15*	0.024	0.07
4	184.774	184.81	12	*ACSL1*	0	1
5	56.558	56.566	6	*C5orf67*	0.017	0.2
5	102.257	102.287	3	*SLCO4C1*	− 0.005	0.7
6	20.529	20.989	42	*CDKAL1*	− 0.001	0.9
6	*31.139*	*32.629*	*32*	*HLA region*	*− 0.049*	*3e−04*
6	126.336	126.76	14	*CENPW*	0.002	0.9
7	13.846	15.026	33	*DGKB*	0.004	0.8
7	28.102	28.217	21	*JAZF1*	0.031	0.02
7	157.184	157.239	2	*UBE3C*	0.002	0.9
8	41.662	41.677	3	*ANK1*	− 0.006	0.7
8	117.171	117.225	9	*SLC30A8*	0.01	0.4
8	144.284	144.328	12	*BOP1*	0.009	0.5
9	22.003	22.136	14	*CDKN2B*	0.005	0.7
9	81.68	81.697	5	*TLE1*	0.007	0.6
9	123.351	123.361	6	*CRB2*	0.001	1
10	12.26	12.272	2	.	0.003	0.8
10	79.181	79.205	16	*ZMIZ1*	0.024	0.07
10	92.197	92.733	62	*CPEB3*	− 0.005	0.7
10	112.97	113.095	18	*TCF7L2*	− 0.011	0.4
10	122.356	123.034	10	*ACADSB*	0.01	0.4
11	2.639	2.837	23	*COX6CP18*	0.011	0.4
11	17.347	17.4	8	*ABCC8*	− 0.005	0.7
11	43.731	43.857	7	*HSD17B12*	− 0.015	0.3
11	65.589	65.896	7	*AP5B1*	− 0.022	0.1
11	92.941	92.976	9	*MTNR1B*	− 0.002	0.9
12	4.182	4.267	2	*CCND2-AS1*	0.017	0.2
12	*27.766*	*27.812*	*9*	*KLHL42*	*0.048*	*3e−04*
12	33.228	33.23	1	.	0.013	0.3
12	65.792	65.827	4	*HMGA2*	0.015	0.3
12	120.614	121.034	35	*ACADS*	− 0.003	0.8

Table 6 Regressions of age at onset on regional genotypic scores for type 2 diabetes (*Continued*)

Chrom	Start (Mb)	End (Mb)	Num SNPs	Nearest gene	Coefficient	*p* value
13	80.131	80.187	5	.	0.003	0.8
15	61.826	62.123	22	*C2CD4A*	− 0.004	0.8
15	77.092	77.615	22	*HMG20A*	− 0.022	0.1
15	90.961	91.024	11	*PRC1*	0.029	0.03
16	53.764	53.811	14	*FTO*	0.005	0.7
16	75.201	75.269	16	*BCAR1*	0.033	0.01
16	81.491	81.501	2	*CMIP*	0.008	0.5
17	3.981	4.37	15	*ANKFY1*	0.01	0.5
17	9.862	9.903	13	*GLP2R*	0.009	0.5
17	48.88	49.018	16	*ATP5G1*	− 0.009	0.5
18	7.062	7.077	5	*LAMA1*	− 0.002	0.9
18	60.064	60.246	15	*RNU4-17P*	0.015	0.3
19	44.889	45.811	12	*APOC1*	0.022	0.09
22	29.74	30.214	16	*ASCC2*	0.01	0.4
22	49.963	50.015	11	*IL17REL*	0.002	0.9
.	.	.	52	Residual genome-wide	0.022	0.1

Table 7 Regressions of log C-peptide on regional genotypic scores for type 1 diabetes

Chrom	Start (Mb)	End (Mb)	Num SNPs	Nearest gene	Coefficient	*p* value
1	39.526	39.585	2	*BMP8A*	0.018	0.1
1	63.643	63.66	3	*PGM1*	0.01	0.3
1	113.286	113.994	28	*PTPN22*	− 0.027	0.01
1	172.706	172.71	1	.	0.024	0.03
1	206.767	206.784	2	*IL10*	− 0.003	0.8
2	19.704	19.704	1	.	− 0.007	0.5
2	*100.13*	*100.254*	*10*	*AFF3*	*− 0.029*	*0.009*
2	162.254	162.404	4	*IFIH1*	− 0.016	0.2
2	203.829	203.93	13	*CTLA4*	0.003	0.8
4	122.078	122.64	9	*IL21*	0.017	0.1
4	165.65	165.653	1	.	− 0.011	0.3
5	168.799	168.808	1	*SLIT3*	0.013	0.3
6	*25.082*	*33.885*	*941*	HLA residual	*− 0.06*	*6e−08*
6	*25.082*	*33.885*	*941*	HLA serotype	*− 0.071*	*4e−10*
6	90.104	90.293	7	*BACH2*	− 0.021	0.06
6	126.334	126.556	3	*CENPW*	0.018	0.1
7	50.399	50.971	6	*IKZF1*	0.015	0.2
9	4.283	4.296	8	*GLIS3*	− 0.014	0.2
10	6.038	6.14	13	*IL2RA*	− 0.026	0.02
10	33.13	33.137	1	*NRP1*	− 0.02	0.07
11	*2.08*	*2.291*	*13*	*INS*	*− 0.052*	*3e−06*
11	119.966	119.966	1	.	− 0.01	0.4
12	55.971	56.125	12	*ERBB3*	− 0.002	0.9
12	110.922	112.755	38	*SH2B3*	− 0.011	0.3
14	68.797	68.797	1	.	− 0.011	0.3
14	98.033	98.033	1	.	0.001	0.9
15	78.939	78.947	2	*CTSH*	0.007	0.5
16	10.946	11.342	36	*CLEC16A*	− 0.014	0.2
16	28.29	28.62	8	*IL27*	− 0.008	0.4
16	75.213	75.262	3	*BCAR1*	0.007	0.5
17	39.82	40.668	13	*ORMDL3*	0.008	0.5
17	45.997	45.997	1	*MAPT*	0.005	0.6
18	12.747	12.877	8	*PTPN2*	0.002	0.9
18	69.863	69.872	2	*CD226*	0.012	0.3
19	46.716	46.716	1	*PRKD2*	− 0.009	0.4
19	48.704	48.704	1	*FUT2*	− 0.003	0.8
20	1.63	1.695	4	*RN7SL561P*	0.005	0.7
20	9.424	9.424	1	*PLCB4*	0.025	0.02
21	42.416	42.428	5	*UBASH3A*	0.006	0.6
22	29.715	30.765	17	*ASCC2*	0.007	0.5
22	37.148	37.195	3	*RAC2*	0.004	0.7
.	.	.	29	Residual genome-wide	− 0.008	0.4

Table 8 Regressions of log C-peptide on regional genotypic scores for type 2 diabetes

Chrom	Start (Mb)	End (Mb)	Num SNPs	Nearest gene	Coefficient	*p* value
1	39.147	39.57	6	*BMP8A*	0.013	0.2
1	213.972	213.99	15	*PROX1*	0.011	0.3
1	219.479	219.615	14	*ZC3H11B*	0.007	0.5
2	43.222	43.623	67	*LINC01126*	0	1
2	60.307	60.372	17	*MIR4432HG*	0.007	0.5
2	65.415	65.457	3	*SPRED2*	0.013	0.3
2	160.234	160.4	18	*ITGB6*	− 0.014	0.2
2	226.133	226.317	17	.	0.004	0.7
3	11.986	12.372	14	*ACTG1P12*	0.017	0.1
3	22.936	23.441	4	*RPL24P7*	0.011	0.3
3	64.714	64.744	7	*ADAMTS9-AS2*	− 0.013	0.2
3	123.217	123.391	7	*ADCY5*	0.003	0.8
3	185.556	185.83	20	*IGF2BP2*	0.023	0.04
4	6.263	6.323	31	*PPP2R2C*	0.016	0.2
4	94.08	94.215	9	*HMGB3P15*	0.003	0.8
4	184.774	184.81	12	*ACSL1*	0.006	0.6
5	56.558	56.566	6	*C5orf67*	− 0.001	0.9
5	102.257	102.287	3	*SLCO4C1*	− 0.012	0.3
6	20.529	20.989	42	*CDKAL1*	0.024	0.03
6	*31.139*	*32.629*	*32*	*HLA region*	*− 0.073*	*5e−11*
6	126.336	126.76	14	*CENPW*	0.026	0.02
7	13.846	15.026	33	*DGKB*	− 0.004	0.7
7	*28.102*	*28.217*	*21*	*JAZF1*	*0.03*	*0.007*
7	157.184	157.239	2	*UBE3C*	0.009	0.4
8	41.662	41.677	3	*ANK1*	0.008	0.5
8	117.171	117.225	9	*SLC30A8*	0.007	0.5
8	144.284	144.328	12	*BOP1*	0	1
9	22.003	22.136	14	*CDKN2B*	0.028	0.01
9	81.68	81.697	5	*TLE1*	0.002	0.9
9	123.351	123.361	6	*CRB2*	0.025	0.02
10	12.26	12.272	2	.	0.016	0.1
10	79.181	79.205	16	*ZMIZ1*	0.011	0.3
10	92.197	92.733	62	*CPEB3*	0.016	0.1
10	*112.97*	*113.095*	*18*	*TCF7L2*	*0.03*	*0.008*
10	122.356	123.034	10	*ACADSB*	− 0.004	0.7
11	2.639	2.837	23	*COX6CP18*	0.023	0.04
11	17.347	17.4	8	*ABCC8*	− 0.004	0.7
11	43.731	43.857	7	*HSD17B12*	0.022	0.04
11	65.589	65.896	7	*AP5B1*	0.006	0.6
11	92.941	92.976	9	*MTNR1B*	− 0.011	0.3
12	4.182	4.267	2	*CCND2-AS1*	− 0.013	0.2
12	27.766	27.812	9	*KLHL42*	− 0.016	0.2
12	33.228	33.23	1	.	− 0.001	0.9
12	65.792	65.827	4	*HMGA2*	0.016	0.1
12	120.614	121.034	35	*ACADS*	0.005	0.6

Table 8 Regressions of log C-peptide on regional genotypic scores for Type 2 diabetes (*Continued*)

Chrom	Start (Mb)	End (Mb)	Num SNPs	Nearest gene	Coefficient	*p* value
13	80.131	80.187	5	.	0.011	0.3
15	61.826	62.123	22	*C2CD4A*	− 0.001	0.9
15	77.092	77.615	22	*HMG20A*	0.022	0.04
15	90.961	91.024	11	*PRC1*	− 0.006	0.6
16	53.764	53.811	14	*FTO*	− 0.006	0.6
16	75.201	75.269	16	*BCAR1*	− 0.006	0.6
16	81.491	81.501	2	*CMIP*	− 0.001	0.9
17	3.981	4.37	15	*ANKFY1*	− 0.002	0.9
17	9.862	9.903	13	*GLP2R*	− 0.017	0.1
17	48.88	49.018	16	*ATP5G1*	0.016	0.2
18	7.062	7.077	5	*LAMA1*	− 0.005	0.7
18	60.064	60.246	15	*RNU4-17P*	0.027	0.02
19	44.889	45.811	12	*APOC1*	0.002	0.8
22	29.74	30.214	16	*ASCC2*	0.003	0.8
22	49.963	50.015	11	*IL17REL*	− 0.012	0.3
.	.	.	52	Residual genome-wide	0	1
